# Predictors for Clinical Outcomes Related to Upper Extremity Musculoskeletal Disorders in a Healthy Working Population

**DOI:** 10.3390/ijerph18179171

**Published:** 2021-08-31

**Authors:** Oliver Lotter, Tobias Lieb, Jochen Molsner, Viktor Breul

**Affiliations:** 1 Department of Plastic, Aesthetic, Hand and Reconstructive Surgery, Academic District Hospital, Zeppelinstrasse 21, 78532 Tuttlingen, Germany; 2 Office for Occupational and Hand Therapy, Neuhauser Strasse 85, 78532 Tuttlingen, Germany; tobiaslieb@gmx.de; 3 IAS-Group for Occupational Health Management, Koenigstrasse 6, 78532 Tuttlingen, Germany; Jochen.Molsner@ias-gruppe.de; 4 Department of Medical Scientific Affairs, Aesculap AG, Am Aesculap Platz, 78532 Tuttlingen, Germany; viktor.breul@aesculap.de

**Keywords:** work-related musculoskeletal disorders (WMSDs), upper extremity, repetitive work, surgical device mechanics, DASH score, Purdue Pegboard Test, multiple analysis, correlation, predictors

## Abstract

A wide range of endpoints and methods of analysis can be observed in occupational health studies in the context of work-related musculoskeletal disorders (WMSDs). Comparison of study results is therefore difficult. We investigated the association between different clinical endpoints and the presence of upper extremity WMSDs in a healthy working population. Furthermore, the influence of socio-demographic, work-related, and individual predictors on different endpoints was examined. Two self-administered questionnaires were distributed to 70 workers and employees. In addition, a standardized physical examination and an industry test were performed in this cross-sectional study. Correlations between WMSDs and clinical endpoints were analyzed with the Spearman method and prediction ellipses. Multiple regression models were used to study the strength of associations with a pre-defined set of potential influencing factors. The prevalence of WMSDs was 56% (39/70). Disabilities of Arm, Shoulder, and Hand (DASH) score/pain under strain showed the strongest correlations with WMSDs. When analyzing the correlation between WMSDs and pre-selected predictors, none of the predictors could be identified as a risk factor. The DASH score remains a close candidate for best surrogate endpoint for WMSDs detection. Standardized analysis methods could improve the methodological quality of future occupational health studies.

## 1. Introduction

Work-related musculoskeletal disorders (WMSDs), also known as cumulative trauma disorders (CTD), repetitive strain injuries (RSI), or occupational overuse syndromes (OOS), account for more than 48% of work-related disorders [1,2]. The overall global prevalence for such conditions ranges from 4.0% to 30%, increasing with age, and the annual prevalence lies between 0.14% and 14.9% across different industries and work processes [3,4,5,6]. Treatment costs for WMSDs are estimated at 1.3% of the US gross national product and between 0.5% and 2.0% in Scandinavian countries [7,8].

WMSDs are of multifactorial origin. The work environment contributes significantly to the condition and/or the condition is aggravated by the work activity [9]. Such disorders are caused by the accumulation of microtraumatic events in the musculoskeletal system over a long period of time and represent a broad spectrum of inflammatory and degenerative diseases, jeopardizing the quality of life and functional capacity of those affected [10,11]. They are a significant occupational health problem among industrial and clerical workers with a strong medical, economic, and social impact in terms of absences due to sickness and early retirement, cost of medical care, low productivity, and personal suffering [12]. These disorders represent one of the greatest work-related challenges of our time, and they have a great impact on individuals and society.

Various physical/biomechanical (e.g., repetitive hand motion), psychosocial (e.g., decision latitude at work), organizational (e.g., night shifts), and genetic (e.g., gender) factors have been linked to the development of WMSDs in numerous occupational settings and specific industries [13,14,15]. Studies to date have only been able to consider some of these factors simultaneously, making it difficult to build a closed picture of the interactions between them [16].

The aim of this analysis was to investigate the association between the presence of upper extremity WMSDs in a healthy working population and different clinical endpoints that are likely to be measured in the clinical routine. Usually, these endpoints only represent more or less useful surrogates for an actual health problem, whose roles should be more clearly determined in order to improve the efficiency of the research. This analysis is also intended to investigate a set of potentially predisposing socio-demographic, work-related, and individual characteristics (“risk factors” or “predictors”) for WMSDs and related clinical endpoints.

## 2. Materials and Methods

### 2.1. Study Population

The study was conducted at the headquarters and main production site of Aesculap AG, Tuttlingen, Germany, with about 3500 workers and employees. This company is the world market leader in the field of surgical instruments. The study population was divided into three groups based on their occupational activities: group I = grinding and polishing, characterized by repetitive and forceful exertions (“grinding”), group II = inspection and packaging, characterized by repetitive exertions without force (“packaging”), and group III = all other white-collar and blue-collar employees as a cross-section of the company without exposure to grinding or packaging as a control group (“control”). Approval of the study was obtained from the Ethics Committee of the Baden-Wuerttemberg Medical Association, Jahnstrasse 40, 70597 Stuttgart, Germany (project number F-2017-005).

### 2.2. Study Design and Data Collection

Random samples of active white-collar and blue-collar workers were drawn from the three groups using statistic software and were recruited between September 2017 and March 2018. No incentives were offered, and participation decision was met voluntarily on invitation by signing the informed consent. The following eligibility criteria were applied according to our previous publication [17]: Age < 18 or > 65 years; employment in the respective workplace for less than 5 years; currently not on sick leave; no absence from work due to upper extremity pain for more than 2 weeks within the last 3 months; and none of the following conditions: cervical spine syndrome or herniated intervertebral disc, shoulder pain radiating into the forearm, debilitating congenital malformation of the upper extremity, rheumatoid conditions including fibromyalgia, previous upper extremity surgery due to nerve entrapment syndrome(s), and/or chronic musculoskeletal disorders, such as tennis elbow; golfer’s elbow; or tenosynovitis of the flexor and/or extensor tendons, including trigger finger and de Quervain’s disease, and three or less unanswered items in the DASH disability/symptom questionnaire.

The participants were asked to fill in two standardized, self-administered questionnaires. The first questionnaire obtained demographic and personal data, considered as predictors in the analyses, such as sex, handedness, secondary occupation, sporting and physical hobbies, age, height and weight (body mass index), employment level, and years of service. Participants completed the questionnaires at the company’s health center independently and without assistance.

### 2.3. Clinical Endpoints and Signs for WMSDs

#### 2.3.1. Disabilities of the Arm, Shoulder, and Hand (DASH) Score

As a second questionnaire, the validated DASH outcome measure was applied to assess physical function and symptoms [18,19]. The results of this 30-item questionnaire were used to calculate a scale score ranging from 0 (no disability) to 100 (most severe disability).

#### 2.3.2. Visual Analog Scale (VAS)

VAS is an instrument to measure the intensity of pain [20]. A Likert scale between 0 (no pain) and 10 (maximal pain) was used to collect the subjective evaluation for pain at rest and pain under strain in the form of a self-reporting measure. Results for pain at rest and pain under strain were analyzed as independent continuous endpoints.

#### 2.3.3. Range of Motion (ROM)

ROM measurements of active wrist joint mobility of both hands were performed using a goniometer in the three planes extension/flexion (E/F), supination/pronation (S/P), and ulnar/radial abduction (U/R). The measurements were recorded according to the neutral zero method [21]. The overall ranges in degrees were calculated for each plane. The primary/non-primary hand and the plane were considered as repeated measures variables in variance analyses.

#### 2.3.4. Grip Strength

Grip strength is the measurement of the maximum hand force using the Jamar dynamometer in three consecutive passes per side [22]. The measurement unit is kg. The primary/non-primary hand and the consecutive pass number were also considered as repeated measures variables. Grip strength is the accepted method of measuring the gross motor skills of the hand.

#### 2.3.5. Purdue Pegboard (PPB) Test

The neurophysiological PPB Test was performed to determine the dexterity of the participants [23]. As a former industrial test, it now serves primarily to assess disabilities and limitations. The pegboard consists of a board with two parallel rows of 25 holes, into which cylindrical metal pegs are placed by the examinee. The test involves a total of four trials [24]. The subsets for preferred, non-preferred, and both hands require the test person to place the pins in the holes as quickly as possible, with the score being the number of pins placed in 30 s. Purdue Pegboard trial number four was chosen as the representative clinical endpoint as it summarizes trial numbers 1 to 3 well by adding them up, and therefore should show the potential differences more clearly.

#### 2.3.6. Subjective Complaints

The presence of subjective complaints regarding the upper extremities of a test person was assessed in the form of a yes/no type of self-reported question. Subjects were asked about general complaints (pain, restriction of movement, and/or numbness of the upper extremity) with the request for differentiation into complaints of the elbow, forearm, wrist, and/or hand [25].

#### 2.3.7. Clinical Signs of Work-Related Musculoskeletal Disorders

Musculoskeletal diagnoses were measured via a structured physical examination of the elbow, forearm, wrist, and hand by one single hand surgeon. The examiner was blinded to the questionnaire responses of the test persons. The diagnoses of De Quervain’s tenosynovitis, lateral epicondylitis, and nerve entrapment syndromes, including carpal tunnel syndrome (median nerve), cubital tunnel syndrome (ulnar nerve), and Guyon’s canal syndrome (ulnar nerve) were made based on pathognomonic clinical signs for upper extremity pathologies, after selection by a multidisciplinary team consisting of an occupational physician, hand surgeon, and occupational therapist [23]. These included pressure pain on the radial side of the wrist along with Finkelstein’s test for De Quervain’s tenosynovitis, lateral epicondyle pain, Maudsley’s test for lateral epicondylitis, and the combination of Hoffman–Tinel sign and static two-point discrimination (2-PD) for finger sensibility for nerve entrapment syndromes, adding Phalen’s test specifically for Carpal tunnel syndrome [26,27,28,29,30]. The presence of any of the above diagnoses in a study participant was considered to be a WMSDs diagnosis, which served as the gold standard for correlation analyses with other clinical endpoints and predictors.

### 2.4. Statistical Analysis

The study sample size was initially calculated to provide a sufficient power for the proof that occupational groups did not differ too much (hypothesis of clinical equivalence) in regard to the DASH score [17]. This hypothesis could be confirmed by primary analysis.

In the current analysis, correlations between variables were assessed using Spearman correlation coefficients and their *p*-values. Prior to the calculation of correlation coefficients, bivariate scatter plots were visually examined in order to investigate interrelationships between the clinical endpoints. Prediction ellipses were applied to the scatter plots. Because the ellipse is centered at the two-dimensional mean and expanded to cover the maximal part of the data points, it can visually indicate the strength of interrelation as well as outliers in the data. A stretched tilted ellipse indicates highly correlated variables, whereas an ellipse that is nearly circular indicates little correlation.

Statistically, the confidence ellipse visualizes the Pearson correlation coefficient, which is the parametric counterpart of the Spearman coefficient. It seems to be a good first step to show the data distribution and the linearity-based correlation strength and direction. The confidence ellipse works perfectly when the correlation between two variables is linear, but is also applicable to dichotomous variables. Deviations from linearity, outliers, or even the impossibility to, a priory, determine whether the linearity assumption is met, make the Spearman correlation coefficient more reliable in explorative settings.

Scatter Plot Matrices with prediction ellipses were used in order to simultaneously visualize bivariate distributions and Pearson correlations in sets of variables.

A set of potential predictors influencing clinical endpoints (i.e., independent variables or effects) was chosen based on the clinical considerations, previous studies, and literature data [9]. Six variables were selected for this role:

- Gender (female/male)
- Body mass index (BMI)
- Occupation group (grinding/packaging/control)
- Secondary occupation and/or physical hobbies
- Age
- Years in service

A multiple regression modeling of clinical endpoints was applied in order to identify their significant predictors, based on the *p*-value of the effect. Linear or logistic regression was used, dependent on a respective continuous or dichotomous type of the endpoint variable.

Some endpoints in our selection enclose repeated measurements in the same subject, such as left- and right-hand measurements of hand force or range of motion. Additionally, the repetition scheme may have included three subsequent attempts with each arm (hand force) or a recording of three dimensions (E/F, S/P, U/R) for range of motion. Adequate use of such repeated data structures required consideration of more sophisticated repeated measurement modeling methods, taking into account measurements that belong to the same participant when analyzing variance. Repeated measurements methods are superior to just using the mean of the three attempts, as it avoids loss of information and statistical power.

Considering handedness (dominant hand) was more challenging than it appears. The study population included four types, as shown in Table 1.

For analytic purposes, left- and right-handed subjects were put together with their corresponding mixed types.

Measurements referring to the right or left body side were analyzed as referring to the primary or to the non-primary hand in order to evaluate the effect of handedness. Instead of distinguishing between right- and left-handedness, we used the parameter dominant/non-dominant hand for further analysis.

Mean values and standard deviations (SD) in brackets were described with approximately normally distributed continuous data. Median and interquartile ranges in brackets were shown for non-normally distributed continuous data [31]. We calculated absolute and relative frequencies for categorical variables. Numerators and denominators of the calculations were always given in parenthesis when reporting percentages in categorical data.

As the study sample of 70 individuals might lack power to detect small effects, the regression results were considered with caution. The classic 5.0% significance level did not seem appropriate for exploration purposes, so effects showing higher *p*-values were closely considered. A threshold of 0.1 was applied in this analysis as it seemed to be useful to show the correlations between the endpoints in a comprehensible way, although any other threshold might also be appropriate. This procedure followed our aim to understand the contributors to clinical endpoints for future studies rather than providing evidence for correlations in our study sample.

SAS software version 9.4 with Enterprise Guide 7.1 GUI (SAS Institute, Cary, NC, USA) was used for analyses.

## 3. Results

### 3.1. Recruitment and Baseline Characteristics

The total population consisted of 63 persons in group I, 208 in group II, and 2501 in group III. Random samples were drawn from these groups after completion of the questionnaires and proving eligibility, so that a total of 70 individuals (grinding *n* = 20, packaging *n* = 24, control *n* = 26) were included in the study. The participants were predominantly men (67% (47/70)) and right-handed individuals (83% (55/70)). Only a few had a secondary occupation (9.0% (6/70)), and 61% (43/70) reported having sporting or physical hobbies. The three groups had comparable demographic data with regard to age (42.1 (±12.2) years), body mass index (BMI) (26.2 (±5.0) kg/m^2^), full employment level (91% (64/70)), and years of service at the company (16.1 (9 to 28) years). For flowcharts and detailed demographic data of the individual groups, please refer to our previous publication [17].

### 3.2. Clinical Endpoints

The DASH score, clinical parameters, and the Purdue Pegboard (PPB) Test score are shown in Table 2. Our mean scores are in good agreement with the normative DASH score [32]. Three DASH questionnaires were excluded from the analysis because of incompleteness. We did not expect any relevant differences between the grip strength of the right and left hands; therefore, we used the parameter dominant/non-dominant hand for further analysis. When comparing range of motion in three levels and grip strength with reference values from a healthy population, subdivided according to sex and age group, normal or below-average values were found [33,34,35]. This was also the case with the PPB test [36].

Subjective complaints (i.e., symptoms) were present in 47% (33/70) of the participants, and pathognomonic clinical signs for upper extremity WMSDs (i.e., diagnoses) at the elbow, forearm and/or wrist (trigger finger, Finkelstein’s test, Maudsley’s test, Hoffman–Tinel sign, and Phalen’s test) were found in 56% (39/70) of the participants (Table 3). Bilateral manifestation was present in 34% (24/70), and 14% (10/70) of the participants had two or more different pathologies in the ipsilateral limb. In the case of a positive Hoffmann–Tinel sign, 91% (21/23) of these were located at the medial elbow as a sign of ulnar tunnel syndrome.

### 3.3. Correlation of WMSDs with Other Clinical Endpoints

**Figure 1:**
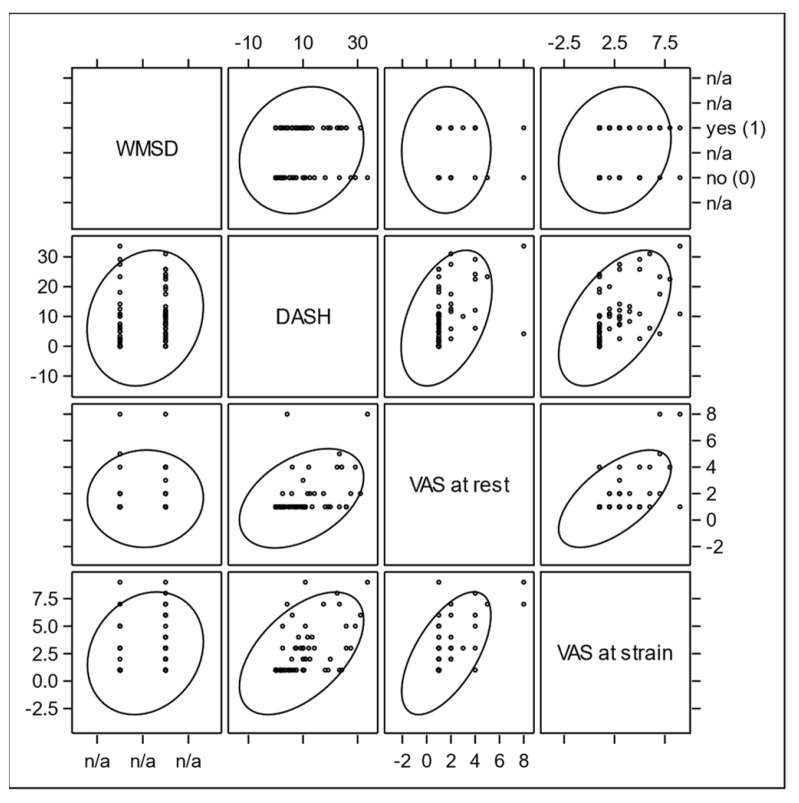
Scatter plot matrix showing the bivariate distributions for all pair-wise combinations of WMSDs, DASH, VAS at rest, and VAS at strain.

**Figure 2:**
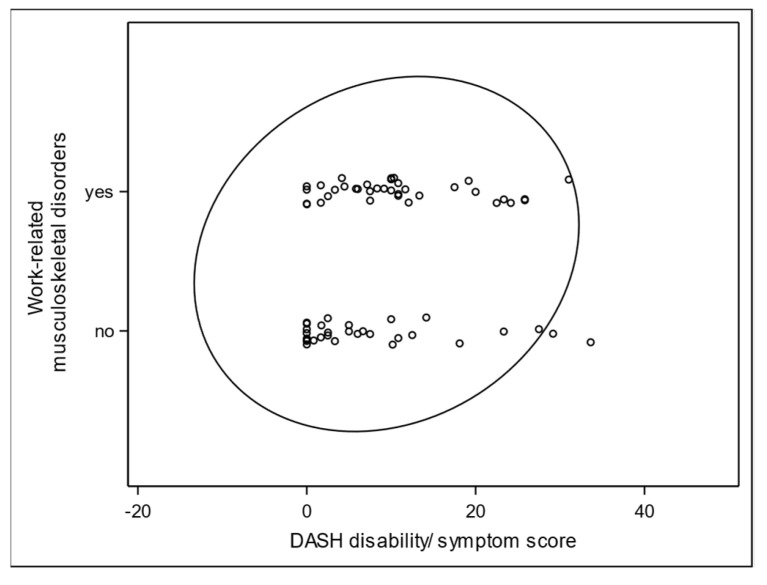
Prediction ellipse for graphical representation of the correlation between the DASH score and WMSD.

The relationship between WMSDs and the DASH score is shown as an example using a scatter plot with a prediction ellipse (Figure 1 and Figure 2) [37]. Further correlations between grip strength and ROM E/F, and between VAS at rest and under strain, DASH, and subjective complaints became apparent.

Bivariate correlation coefficients between clinical endpoints and the diagnosis of WMSDs are shown in Table 4. Only two of the collected clinical endpoints were associated with a *p*-value below 0.1: the DASH score and VAS under strain. Both endpoints were positively correlated with WMSDs, meaning that higher scores are associated with a WMSDs diagnosis. These results also make sense from a clinical point of view because both DASH scores and VAS associate higher scores with worse outcomes.

### 3.4. Multiple Regression Analyses of Predictors

The relationship between the pre-specified predictors and the occurrence of WMSDs was investigated through the analysis of a binary logistic regression (Table 5). None of these factors seem to be a risk factor for WMSDs, as the minimal *p*-value was 0.14.

The respective analyses following the same layout were performed using other clinical endpoints, with a linear instead of logistic regression applied in the case of continuous endpoints. Results are shown in Table 6. Only the combinations with a *p*-value of *p* ≤ 0.1 are listed.

In addition to known and obvious correlations such as gender dependence for DASH with higher values in women, or higher grip strength in men, there was a negative correlation between ROM and years in service, more frequent subjective complaints of the upper extremities with increasing age, pain under strain on the VAS at a higher BMI, and higher grip strength when secondary occupation and/or physically demanding hobbies were performed [38]. A statistical effect may be observed in Table 5 with by far the lowest *p*-values concentrated in the analysis of grip strength and, to some extent, of ROM.

## 4. Discussion

We chose to survey a population of actively employed surgical device mechanics and compared them with a group of employees believed not to be exposed to repetitive hand and arm movements to such a large extent.

The overall prevalence of subjective upper extremity complaints (i.e., symptoms) was 47% (33/70). Eight out of 20 (40%) grinders, 14/24 (58%) packers, and 11/26 (42%) people in the control group reported such symptoms. One or more upper extremity WMSDs (i.e., diagnoses) at the elbow, forearm, and/or wrist were found in 56% (39/70), of which 12/20 (70%) were grinders, 14/24 (54%) were packers, and 13/26 (42%) were control persons. These results are consistent with previous studies showing a prevalence rate of symptoms between 21% and 71% in the study group and between 6.0% and 50% in the control group [5,39,40,41,42,43,44,45,46,47,48,49,50]. With regard to diagnoses, existing studies report a prevalence between 21% and 56% in the study group compared to 5.0% to 22% in the control group [5,39,41,42,43,45]. Within the framework of the standardized clinical examination, we applied rather “softer” criteria, as recommended by Vikari-Juntura [51]. This might explain the higher detection of WMSDs via examination as compared to the lower number of subjective complaints reported in the questionnaire. An above average co-occurrence of medial epicondylitis (golfer’s elbow) and nerve entrapment at the medial elbow (cubital tunnel syndrome) was found in 30% of our test persons and 54% of the individuals with upper extremity complaints in this cohort [52,53,54].

Study designs and research methods used to collect and process data via WMSDs are extremely heterogeneous. Our literature search revealed that, out of 262 original research articles, only about 4.0% of the studies contained a clinical examination of the individuals with or without structured questionnaires/interviews including a control group [17]. Most studies were based on self-administered questionnaires and results from health insurance databases without a control group.

The clinical endpoint values of our study population were largely consistent with the reference values of the general population, but in some cases (grip strength, PPB tests) also showed below average values. This is surprising and contradicts the study situation, as our cohort tends to have an above-average physical load [55]. The reasons for this could be mechanical support, shorter working hours, and a historical shift in populations’ reference values without the first two points mentioned.

A closer look into the scatter plot revealed an obvious floor effect in the DASH distribution (Figure 1). DASH values of probands with WMSDs concentrated more in the ten-point range, whereas the “no WMSDs” group very often had a DASH score of 0. This caused the slight tilt of the ellipse, correlating with the Pearson correlation coefficient, which, in the first approximation, visualized an interrelation between the DASH score and WMSDs (Figure 2). However, the non-normality contradicted the application of the parametric Pearson regression and caused a switch to the non-parametric Spearman correlation. Its coefficient was indeed much more sensitive to this effect, due to its robustness towards non-normal distribution of the DASH values. For the same reason, the normality assumption was not given for ROM and VAS distributions, so the Spearman method had to be preferred for evaluation of interactions between WMSDs and these endpoints. This particular non-parametric correlation analysis with one dichotomous variable is known as rank-biserial correlation.

In the bivariate analysis, we found a correlation between the DASH score and WMSDs as well as VAS under strain and WMSDs. A simple clinical explanation for this could be that pathology (WMSDs) manifests itself through pain, especially when the hand is used forcefully. This aspect is a common feature of the DASH questionnaire and also manifests itself with VAS under strain. VAS at rest, ROM, grip strength, the PPB Test, and the indication of subjective complaints by the study participants were not suitable to detect WMSDs in our study. However, the question of subjective complaints is a central component of many studies and the basis for their interpretation.

Regarding the upper extremities, the validity of questionnaires for WMSDs has not been clarified, and it is not known how an optimal questionnaire can be constructed and what information can be obtained [56]. A purely technical investigation using measurement data without clinical examination would have the advantage of resource optimization but could not be related to WMSDs either [57]. This is also supported by the relatively weak correlation between measured clinical endpoints and WMSDs in our study. Accordingly, a clinical examination based on a predetermined set of diagnostic criteria remains the gold standard for cross-sectional investigations in order to keep well-defined disorders separate from more diffuse conditions. Although clinical examination is time-consuming and hard for both the subject and the examiner, it seems to be necessary at this time to detect defined WMSDs.

Although the *p*-value has not quite reached the conventional significance level of 0.05 in our study (*p* = 0.056), the correlation we found between WMSDs and the DASH score may not therefore be considered non-existent and could be of interest for the design of future studies [58]. The DASH score as a self-administered, region-specific outcome instrument for upper-extremity disability and symptoms was tested against the gold standard of WMSDs detection (i.e., the clinical examination). To our knowledge, there are no studies focusing on the correlation between WMSDs and the DASH score, whereas this is the case for some upper extremity pathologies other than WMSDs [59]. According to our analysis, the DASH score has the potential to replace the resource-intensive clinical examination as a screening tool. In case of conspicuous DASH scores, the latter could be used in a focused manner for diagnosis, with therapeutic and preventive measures derived from it. The DASH score has shown good validity, reliability, and responsiveness in relation to upper extremity disorders [60]. In comparison, the Nordic Musculoskeletal Questionnaire (NMQ), often used in cross-sectional studies, is a simple validated questionnaire that refers to complaints in nine body parts, including the hand/wrist/elbow [61]. Its content is in no way comparable to the detailed questions of the DASH, with its focus on the upper extremities, and hardly exceeds the yes/no question on subjective complaints in our study. To what extent further questionnaires are suitable for the detection of upper extremity pathologies will be the subject of future studies.

For the endpoint WMSDs, the multiple analysis of our study did not show any independent predictors significant at a 0.1 level. This is partially in contrast to previous studies, in which work-related and socio-demographic characteristics have been determined as predisposing upper extremity disorders [5]. Work-related factors include static postures; excessive force and strain; vibration; repeated pushing, pulling, and lifting; overuse of particular anatomical structures or regions; poor posture or improper positioning; awkward movements; long duration of pressure; rapid work pace; short recovery periods; low decision latitude; years of service; and job satisfaction [39,62,63,64,65]. Socio-demographic characteristics predicting WMSDs include factors such as sex, age, marital status, work experience, body mass index, and physical activities [66,67,68,69,70,71]. For standardization and better comparability of future studies, the authors have selected the predictor variable set following the International Classification of Functioning, Disability, and Health (ICF). This is an internationally recognized classification of health and health-related domains [72]. The scatter plot matrix with prediction ellipses has proven to be a fast graphic analysis and a preliminary stage for a detailed statistical evaluation in our study.

Multiple analyses were also used to examine the independent predictors for other clinical endpoints. Significant positive correlations between ROM and years in service, more frequent subjective complaints of the upper extremity with increasing age, higher VAS under strain with a higher BMI, and higher grip strength with the presence of a secondary occupation and/or physically demanding hobbies are particularly noteworthy. These findings are relevant for future investigations, because the relationships of independent variables to each other may disturb the identification of risk factors for WMSDs, acting as confounders. These relationships should therefore be considered when analyzing any WMSDs-related outcomes.

The literature pool of 262 publications, compiled for the primary publication, was scanned for indications that any multiple regression methods were used for predictor analyses. Only 32 articles (12%) could be identified, indicating that the usage of multivariate analyses is still not common in this type of study.

The very low *p*-values for effects in the analysis of grip strength and range of motion (see Table 6) probably resulted from the increase in power due to the higher number of individual measurements (2 × 3 measurements per subject, i.e., total *n* = 420 in 70 subjects). However, the variance analysis employed in regression models with other simply measured values lacks such an amount of information regarding the variance of the measurements. The additional information gave the analysis of variance more statistical power, i.e., more sensitivity for effects detection.

We intentionally tried to avoid the term “significant” in regard to this analysis, in order not to refer our reported *p*-values to the conventional 5.0% significance level, which may be prone to misinterpretation [58,73]. Using the concept of hypothesis testing in the scientifically accurate way, setting a significance level would require a multiplicity correction for a number of pre-defined tests. Such explicit correction would, on the other hand, take away our flexibility to follow the effects and relations in our data, which contains a complex network of endpoints and their predictors. For this reason, this secondary analysis was clearly explorative. This means that the *p*-values shown in the tables are not referred to any significance level. They rather provide continuous information of how effects are related or ranked according to their strengths. In this respect, Table 3 in the results section has to be considered as identification of two potential candidates for appropriate surrogate measures for WMSDs prevalence. However, real evidence for the adequacy of any of these candidates for WMSDs detection in clinical use has to be generated by a dedicated study.

The DASH score was considered to be the surrogate endpoint of choice for our primary analysis of WMSDs prevalence among medical device manufacturing employees. The current analyses confirm that DASH still has to remain as the closer choice when assessing the WMSDs status of a population.

Regarding the limitations of our study, it should be noted that cross-sectional studies always represent a snapshot, and no statement can be made about the duration of an existing WMSDs. In particular, it is not possible to clearly distinguish between chronic, recurrent, or acute diseases. As in other studies, we also focused on a manageable number of potential risk factors, because an increasing number of predictors increases the probability of false positive effects, especially in smaller samples. This makes it difficult to assess these effects as a whole. Due to the single investigator approach, there is a risk of systematic error for over-sensitive detection of WMSDs, which is indicated by the higher number of diagnoses compared to symptoms in our study. On the other hand, the examination was performed by the same hand surgeon, which may have led to the diagnosis at an earlier stage than in a clinical setting. However, reducing the systematic error by a multiple investigator approach would have brought an inter-observer error into play, arising as a result of different teaching backgrounds and subjective assessments. Repeating the physical examination tests by having two investigators examine the same person was not an option for the authors. A major reason for this is that most test results depend on the announcement of symptoms (pain, numbness, etc.), and participants learn during follow-up examinations, which limits the objectivity of such study designs [57]. The small study sample of 70 individuals might not provide power to detect small effects, so it may be considered another limitation. Even though the cross-sectional design of our study does not permit causal inference, the observed relations provide valuable evidence for further research and policy making. For further limitations with regard to the three occupational activities, we would like to refer to our previous publication [17].

## 5. Conclusions

The methods used to collect, process, and interpret data on WMSDs are extremely heterogeneous, so the comparability between studies is poor. This study evaluated survey methods and assessment tools for the detection of upper extremity WMSDs and its associations in a healthy working population.

While the most frequently used questionnaires focus on subjective complaints that do not seem to be related to WMSDs, the DASH questionnaire could prove to be an efficient screening method. However, the gold standard for the detection of WMSDs, and also for the derivation of prophylactic, therapeutic, and rehabilitative measures, is still the standardized physical examination, based on a predetermined set of diagnostic criteria.

Our analysis has not identified any risk factors for WMSDs in the study data. Possibly, the effects of investigated risks were too small to be detected by our relatively small study sample.

In order to make epidemiological research on upper extremity WMSDs more comparable, a uniform study design in regard to endpoint selection is recommended. We hope that the methodological results of our work will help other researchers to obtain more efficient and consistent tools for the research on upper extremity WMSDs.

## Figures and Tables

**Table 1:** Distribution of handedness in our study population.

	N	%
Total	70	100
Left	6	8.57
Left mixed	6	8.57
Right	55	78.57
Right mixed	3	4.29

**Table 2:** Measured continuous clinical endpoints.

Continuous Clinical Endpoints	N	Measured Values	Reference Values
		Mean (SD)	Mean (SD)
DASH score	67	9.45 (8.96)	10.1 (14.7)
VAS at rest (points)	70	1.61 (1.45)	-
VAS under strain (points)	70	2.53 (2.20)	-
ROM E/F (degrees)	70	125 (13.7)	124 (17)
ROM S/P (degrees)	70	178 (7.08)	165 (-)
ROM U/R (degrees)	70	51.0 (3.36)	60 (10)
Grip strength (kg)—male	70	47.8 (13.56)	54 (7.0)
Grip strength (kg)—female	70	27.3 (4.93)	32 (6.0)
PPB Test (points)	70	41.0 (4.83)	43.2 (5.21) *

DASH = Disabilities of the Arm, Shoulder, and Hand, VAS = pain on Visual Analog Scale, ROM = range of motion, E/F = extension/flexion, S/P = supination/pronation, U/R = ulnar/radial abduction, PPB Test = Purdue Pegboard Test. * Calculated for the mean age category (40–49 years) and for the study population gender ratio (23/70 female) according to [34].

**Table 3:** Measured categorical clinical signs.

Categorical Clinical Signs	Total Participants
Subjective complaints *	47% (33/70)
Trigger finger	10% (7/70)
Finkelstein’s test	10% (7/70)
Maudsley’s test (middle finger test)	14% (10/70)
Hoffman–Tinel sign	33% (23/70)
Phalen’s test	10% (7/70)

* Counts among the clinical endpoints without being a clinical sign, listed in this table for categorical properties.

**Table 4:** Correlation and confidence intervals between WMSDs and other clinical endpoints.

Clinical Endpoint	N	Spearman r
DASH score	67	0.23 (−0.01, 0.45, *p* = 0.056)
VAS at rest	70	0.04 (−0.20, 0.27, *p* = 0.755)
VAS under strain	70	0.26 (0.03, 0.47, *p* = 0.029)
Range of motion E/F	70	−0.01 (−0.24, 0.23, *p* = 0.943)
Range of motion S/P	70	−0.17 (−0.39, 0.07, *p* = 0.168)
Range of motion U/R	70	−0.12 (−0.35, 0.12, *p* = 0.316)
Grip strength	70	0.13 (−0.11, 0.36, *p* = 0.269)
Purdue Pegboard Test	70	0.03 (−0.20, 0.27, *p* = 0.787)
Subjective complaints	70	0.15 (−0.09, 0.37, *p* = 0.213)

DASH = Disabilities of the Arm, Shoulder, and Hand, VAS = pain on Visual Analog Scale, ROM = range of motion, E/F = extension/flexion, S/P = supination/pronation, U/R = ulnar/radial abduction, PPB Test = Purdue Pegboard Test.

**Table 5:** Results of the multiple analysis for the endpoint WMSDs.

Effect/Factor	*p*-Value
Gender (female/male)	0.14
Body mass index (BMI)	0.41
Occupation group (grinding/packaging/control)	0.43
Secondary occupation and/or physical hobbies	0.57
Age	0.96
Years in service	0.99

**Table 6:** Results of the multiple analysis of secondary clinical endpoints.

Basic Endpoint	Effect/Factor	*p*-Value
DASH	Gender (female/male)	0.019
Range of motion	Dimension of movement (E/F, S/P or U/R)	<0.001
	Years in service	0.021
	Gender (female/male)	0.035
Subjective complaints	Age	0.042
	Years in service	0.060
VAS at rest	Body Mass Index	0.091
VAS under strain	Body Mass Index	0.027
Grip strength	Gender (female/male)	<0.001
	Age	<0.001
	Body Mass Index	<0.001
	Secondary occupation and/or physical hobbies	0.041
	Years in service	0.053

DASH = Disabilities of the Arm, Shoulder, and Hand, ROM = range of motion, VAS = pain on Visual Analog Scale, BMI = Body Mass Index, E/F = extension/flexion, S/P = supination/pronation, U/R = ulnar/radial abduction.

## Data Availability

The data presented in this study are available on request from the corresponding author.
